# A virtual reality simulator for training the surgical reduction of patient-specific supracondylar humerus fractures

**DOI:** 10.1007/s11548-021-02470-6

**Published:** 2021-08-07

**Authors:** José Negrillo-Cárdenas, Juan-Roberto Jiménez-Pérez, Joaquim Madeira, Francisco R. Feito

**Affiliations:** 1 Fundación I+D del Software Libre (FIDESOL), Granada, Spain; 2 Department of Computer Science, Graphics and Geomatics Group of Jaén, University of Jaén, Jaén, Spain; 3 Department of Electronics, Telecommunications and Informatics, Institute of Electronics and Informatics Engineering of Aveiro (IEETA), University of Aveiro, Aveiro, Portugal

**Keywords:** Training simulator, Virtual reality, Humerus fractures, Computer-assisted orthopedic surgery (CAOS)

## Abstract

**Purpose:**

Virtual reality has been used as a training platform in medicine, allowing the repetition of a situation/scenario as many times as needed and making it patient-specific prior to an operation. Of special interest is the minimally invasive plate osteosynthesis (MIPO). It represents a novel technique for orthopedic trauma surgery, but requires intensive training to acquire the required skills. In this paper, we propose a virtual reality platform for training the surgical reduction of supracondylar fractures of the humerus using MIPO. The system presents a detailed surgical theater where the surgeon has to place the bone fragments properly.

**Methods:**

Seven experienced users were selected to perform a surgical reduction using our proposal. Two paired humeri were scanned from a dataset obtained from the *Complejo Hospitalario de Jaén.* A virtual fracture was performed in one side of the pair, using the other as contralateral part. Users have to simulate a reduction for each case and fill out a survey about usability, using a five-option Likert scale.

**Results:**

The subjects have obtained excellent scores in both simulations. The users have notably reduced the time employed in the second experiment, being 60% less in average. Subjects have valued the usability (5.0), the intuitiveness (4.6), comfort (4.5), and realism (4.9) in a 1–5 Likert scale. The mean score of the usability survey was 4.66.

**Conclusion:**

The system has shown a high learning rate, and it is expected that the trainees will reach an expert level after additional runs. By focusing on the movement of bone fragments, specialists acquire motor skills to avoid the malrotation of MIPO-treated fractures. A future study can fulfill the requirements needed to include this training system into the protocol of real surgeries. Therefore, we expect the system to increase the confidence of the trainees as well as to improve their decision making.

## Introduction

Traditionally, medical training has been carried out by using cadavers or manikins, where trainees learn under the supervision of an experienced surgeon. This approach is in some cases inefficient due to the considerable number of specimens required to perform a surgical simulation. Moreover, the continuous evolution of medic science forces us to explore and practice safer novel techniques. In fact, surgeons learn faster with simulators than using a classical approach [1–3]. Then, the employment of virtual reality systems allows us to create custom scenarios, adapted to the requirements of a specific intervention, even in rare pathologies [4].

Minimally invasive surgery (MIS) requires the improvement of multiple competences: technical and motor skills, quick acting, knowledge, etc. An intensive training is necessary to acquire them successfully [5]. One example of MIS in orthopedic trauma surgery is Minimally Invasive Plate Osteosynthesis (MIPO). It consists of inserting a stabilization plate by a minimal incision, guided by fluoroscopy or periodical X-ray images [6]. This technique has considerably improved the recovery of the patients, especially in the case of the humerus [7, 8]. However, it frequently causes malrotation of the bone fragments, in spite of being acceptable in most cases [9]. Consequently, we consider the relocation of bone fragments as a crucial task to be trained. Virtual reality simulators allow reproducing specific clinical situations where trainees can solve problems multiple times, receiving objective feedback of their performance. They can explore the initial anatomy of the fracture and internalize the actions to achieve a proper anatomical bone position by repeating the required movements, reducing, in the end, the patient exposition.

In this paper, we present a virtual reality-based simulator for training the reduction of supracondylar humerus fractures adopting a MIPO approach. According to [4], a typical orthopedic trauma surgery is divided into several trainable stages:

- Localization of the surgical area.
- Real reduction.
- Drilling, screwing, needle insertion, and wiring.
- Surgery assessment.

Our proposal is focused exclusively on the *real reduction* step, to train positioning and rotating bone fragments in a MIPO procedure. The paper is organized as follows: section Previous works briefly presents the state-of-the-art of training simulators in medicine and orthopedic trauma surgery; section System description explains the proposed system; section Method details the experiments performed to assess the validity of our proposal; and finally, section Results and discussion concludes the paper.

## Previous works

Patient-specific medical simulation has been implemented by patient body shape adapting to match real patient [10]. Systematic reviews identify a range of haptic and VR interfaces used in simulators for orthopedic surgery [11, 12]. The most popular are related to the hips due to their difficulty and riskiness, especially in the elderly [13]. More concretely, drilling, screw insertion or fixation of fragments are part of the set of tasks that increment the risks associated to this type of surgery. Finite Element Analysis (FEA) has been used in this kind of systems to model bone material properties for fracture or bone drilling [14]. Some companies are aware of risks associated to O&T surgeries, e.g., TraumaVision (Swemac, Linköping, Sweden) is the most relevant solution for simulating hip fractures, and many authors have validated it [15–19]. Similarly, femur fractures are often complicated, requiring specific training and planning. Like hips, they frequently require drilling the bone and inserting plates and screws. The use of simulators with haptic feedback allows us to reproduce realistic sensations [20–24]. These kind of systems can be extended to other long bones that could require a firm fixation, e.g., tibia or radius [25–27].

Another kind of simulators focuses on guiding or assisting in other procedures. For instance, arthroscopy is a common technique to explore the internal part of a joint. It is employed in the so-called arthroscopic surgery, e.g., knee. In that case, systems, such as Virtamed ArthroS (VirtaMed, Zurich, Switzerland) are intended to enrich the skills of the trainees in arthroscopic-based procedures [28–30].

Regarding pure virtual reality simulators, we have found that most works are only focused on non-immersive environments. This is caused by the difficulty to recreate realistic sensations in real-time and lack of performance [4]. In addition, to the best of our knowledge, there are no simulators focusing on the humerus, more concretely on MIPO techniques. However, the techniques employed in above-mentioned systems can be imported to humerus surgical procedures. In this case, a proper orientation of the fragments presents the main difficulty, so a patient-specific simulator is used to train the intervention during a planning stage (prior to the surgery).

## System description

Our proposed system consists of a virtual surgical theater and requires a head mounted display (HMD) and a motion controller with at least six degrees of freedom to allow realistic movements in the virtual world. The description is divided into the following four subsections: firstly, the scenario (section Scenario); next, the interaction paradigm (section Interaction paradigm); afterward, the user interface (section User interface) and finally, the gamification elements (section Score and gamification).

### Scenario

**Fig. 1:**
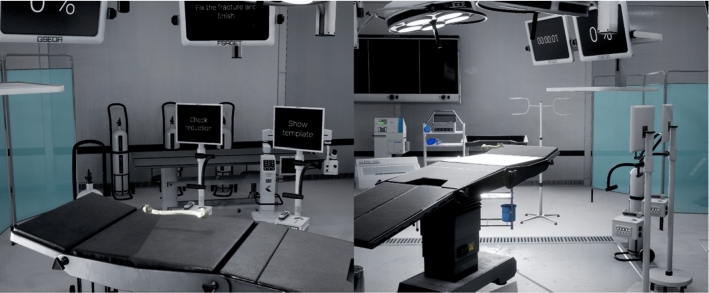
Screenshots of the virtual scenario

The scenario is an essential component of a simulator since it has the ability to place the user in a realistic environment. In this case, we decided to precisely reproduce a operating theater. We inserted a broad variety of equipment, furniture, high-quality illumination, and post-processing effects to ease the user immersion (see Fig. 1).

The specialist initially appears in front of an operating table with the selected fractured bone, used as a starting point of the simulator. He/she needs to examine and fix the case by selecting/moving the fragments.

### Interaction paradigm

The definition of an interaction paradigm requires identifying the set of tasks a user has to perform. Classically, the available actions in a scenario with graphical elements are classified into six groups, according to their requirements [31]:

- *Selection* An element is selected from a set of alternatives.
- *Position* A position of an element is indicated.
- *Orientation* The rotation of an element is modified by the user.
- *Path* The user generates a sequence of positions and orientations over time.
- *Quantification* A value is specified to quantify a measure.
- *Text* The user indicates a text string as a part of the information stored in the computer.

**Fig. 2:**
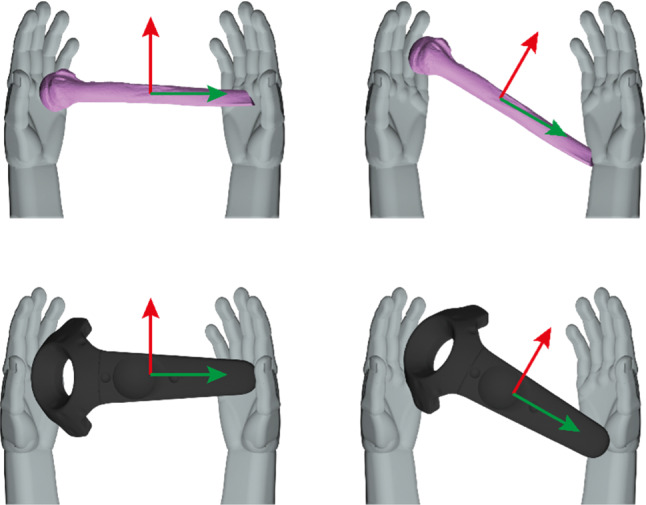
Schematic representation of the interaction paradigm to move the bone fragments [33]. The movements of the remote are directly applied to the virtual object

The proposed prototype requires performing actions related to selection, position, orientation, and path to place bone fragments into their proper anatomical location. As we deal with an immersive VR system, we design the selection as a classical laser-pointer interaction [32]. In other words, the user holds a remote, representing their hands in the virtual environment. Then, a laser is generated from the tip of the remote pointing to its front. Lastly, a ray-casting algorithm determines the selected object. When a fragment is selected, it is automatically attached to the position and orientation of the remote. It allows the free movement of the fragment, like holding it with both hands in the real world [33]. Its position and orientation directly correspond with the remote, as Fig. 2 depicts. Therefore, the *path* task is inherently obtained from the list of movements that the user performs along time.

In addition, the camera follows the basic principle of immersive VR environments, i.e., it is linked to the movement of the head of the user, as the simulator is intended to be used in an HMD-based system. The user can freely move around the operating theater by combining walking and teleportation [34].

Finally, as mentioned above, two interaction paradigms can be distinguished in the proposed simulator:

- Traditional interaction in an immersive VR system. Walk/teleport to move the avatar and select objects with a laser pointer.
- Hand-to-bone movement by holding a remote with both hands.

**Fig. 3:**
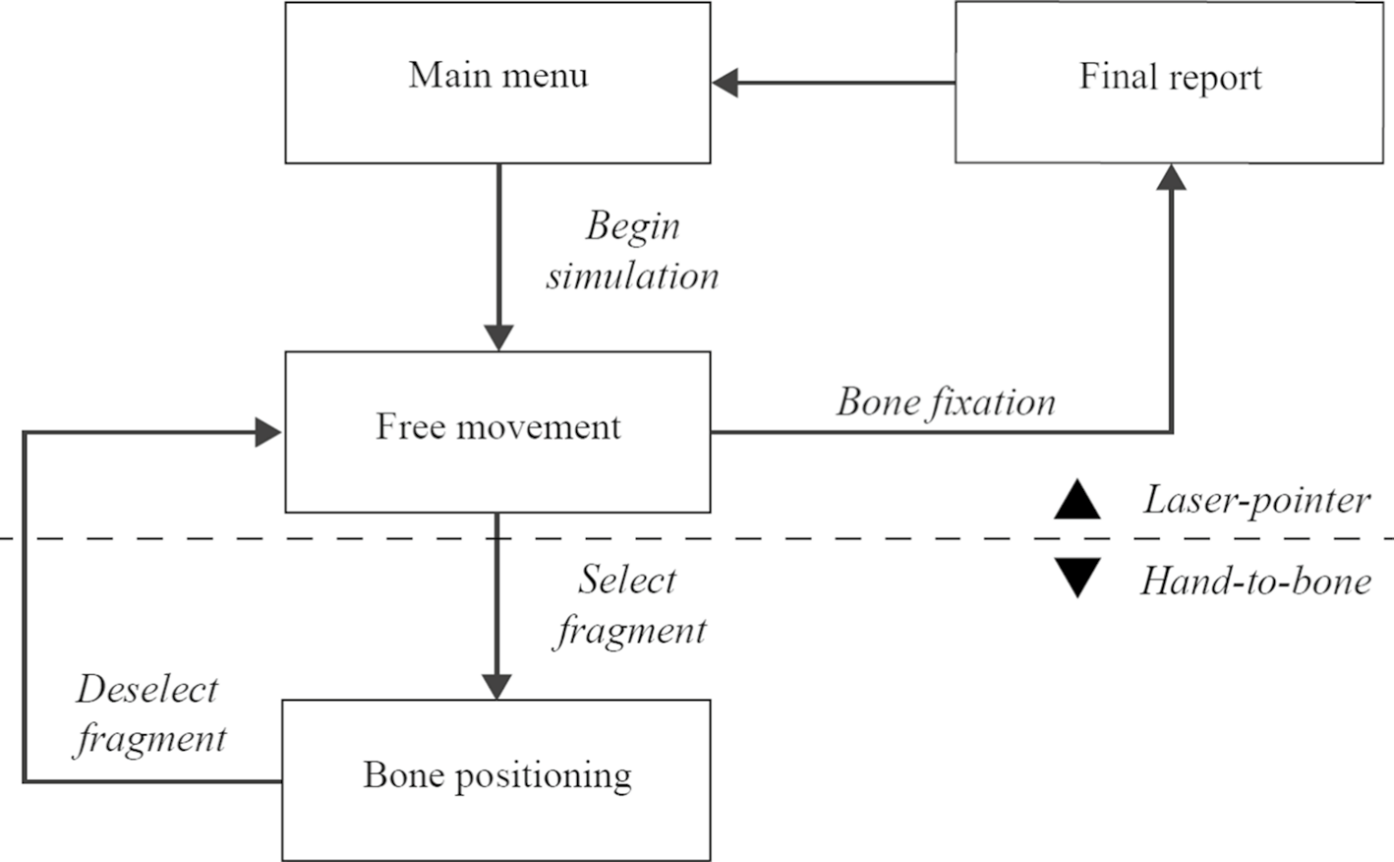
The so-called *flowboard* of the system [35]

The transitions between them are depicted in Fig. 3.

### User interface

Besides a proper placement of bone fragments, the user also performs secondary actions during the simulation. In particular, we have integrated toggle hints, check the quality of the reduction and finish the simulation with feedback information (time elapsed and progress).

The actions are triggered during the simulation by graphical elements integrated in the operating room via a *diegetic* interface [36]. An interface is named diegetic when it is included in the virtual world and can be perceived by the characters. More concretely, they can be triggered by using a laser pointer in surgical panels and screens.

Finally, we have implemented an initial menu to select between a set of cases and a visual final report to summarize the performance of the intervention (Fig. 3).

### Score and gamification

It is well-known that appropriate game-based mechanics allow boosting motivation and solving problems in a more effective way [37, 38]. Orthopedic VR simulators can give a score to assess the skill level of surgeons by giving feedback [39]. They have been shown to improve performance and surgical skills in actual operating rooms [40]. Our goal is to challenge the user with a final score, i.e., the higher the score, the more accurate reduction, and safer intervention.

Firstly, we need to define the meaning of *reduction accuracy*. We have established it through the analysis of bone landmarks of the humerus. An initial landmark detection is carried out in the fractured bone and its contralateral counterpart. This detection is performed by adopting a geometrical approach. We refer the reader to [41] for more details. For each case, we detect the following elements:

- Head.
- Bicipital groove.
- Humeral shaft axis (HSA).
- Trochlea.
- Capitulum.
- Medial and lateral epicondyles.
- Epicondylar axis (ECA).
- Flexion–extension axis (FEA).
- Müller squares [42].
- Other derived measures, such as distances or angles between above-mentioned landmarks.

Once the detection is complete, a direct comparison with its contralateral humerus is performed to obtain the accuracy of the reduction [41]. The ρ coefficient goes from 0 (no reduction) to 1 (perfect reduction).

**Fig. 4:**
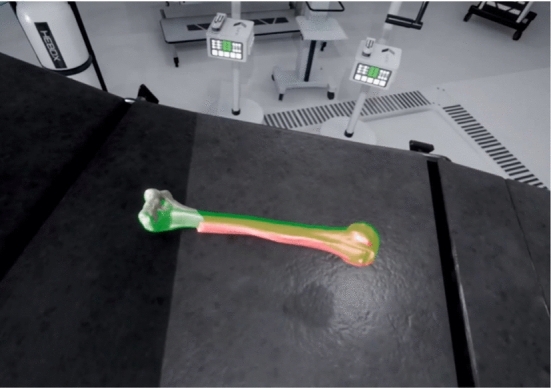
Example of a template of the bone where the user tries to fit the fragments. It corresponds with the contralateral part

Additionally, since the goal of the simulator is to train more confident surgeons, several penalties are imposed to the final score to encourage users to reduce surgical time and excessive radiation:

- *Time elapsed* The more time, the less score. The objective of training is to reduce the intervention time and associated risks.
- *Number of checks* The user can click a button to measure the accuracy of a reduction. In a real scenario, it is equivalent to getting and analyzing a new X-ray image. Therefore, the more checks/X-rays, the less score, to avoid excessive radiation.
- Whether the user uses a template or not (Fig. 4).

As a consequence, the final score is defined by the following formula:

1 $$ score = \left( {k_{1} \rho - k_{2} t - k_{3} n_{c} } \right)*h $$

with *t* being the total time elapsed in seconds, *n*_*c*_ the number of reduction checks and {*k*_1_, *k*_2,_ *k*_3_} a set of constants to adjust the contribution of each component to the final score. Finally, the *h* value represents a factor to be applied in case of using the healthy template, initially *h* = 1.

Finally, besides the previous global indicators displayed after the simulation, a detailed report including the obtained results is sent to the trainee. This document contains specific values regarding the reduction, including a breakdown of the metrics. Each value represents an one-on-one comparison among the elements in the fractured side and the contralateral template [41]. In case of bad indicators, surgeons could consider repeating the simulation before the intervention. It allows them to evaluate possible actions to make the surgery safer and faster, learning from their previous errors.

## Method

This section details the experiments performed to assess the results of our simulator and its contribution to surgical training. The experimental scenario was developed using the Unreal Engine framework (Epic Games, Cary, USA) to design the environment on an HTC Vive headset (HTC, Taoyuan, China). Likewise, The Visualization Toolkit [43] was used to implement the scoring algorithm.

We selected seven users to perform a surgical reduction using the proposed system. They are experienced in using virtual reality in medical contexts, including surgical simulators. Two paired humeri were selected from a dataset obtained from the *Complejo Hospitalario de Jaén*. They were scanned using a GE Brightspeed 16 CT scanner (General Electric, Boston, USA). We have labeled them as *Case 1* (first simulation) and *Case 2* (second simulation) in this paper*.* A virtual fracture was performed in one side of the pair, using the other as its contralateral counterpart. Users have to simulate a reduction for each case. All volunteers performed the sequence of exercises during the experiments. The following information was gathered during the procedure:

- Total time elapsed in seconds.
- Did the user toggle on the hints?
- Number of checks of the quality of the reduction.
- Reduction accuracy.
- Final score.
- Detailed metrics for the final report.

After finishing the simulation, each user had to fill out a survey about usability. It consisted of ten questions using a five-option Likert scale [44], being five the best opinion [45]. The questions are listed below:

- Does the image refresh smoothly when interacting with the application?
- Rate the degree of isolation from the environment during the simulation.
- Rate the realism of the simulation.
- Have you been able to fuse the images of both eyes correctly? (i.e., did you see one single image?)
- Have you felt any dizziness during the simulation?
- Did you feel limited in movement when you were wearing the headset?
- Has the headset been comfortable for you?
- Do you have any previous experience using virtual reality applications in a medical context?
- Is the application intuitive and easy to use?
- Is it easy to learn how to use the application?

Finally, we adjusted the values of the coefficients of Eq. 1 to k_1_ = 1000,* k*_2_ = 0.1, *k*_3_ = 10, and *h* = 0.7 in case of enabling the template.

## Results and discussion

Table 1 shows the statistical results related to the scores and Table 2 details the metrics related to the calculation of the ρ coefficient. The results have been excellent in both cases, but the degree of reduction was slightly higher for the second one. However, we have observed that the users have notably reduced the time employed in the second experiment (60% less time on average). In fact, we noticed that most users avoided toggling on the hints for the second case, being aware of the penalty in the final score. The system has shown a high learning rate and it is expected that the trainees will reach an expert level after several additional runs.

**Table 1:** Descriptive summary of scores

	Average ± SD			Range	
	Case 1	Case 2	Improvement	Case 1	Case 2
Time elapsed	223.43 ± 80.34	94.43 ± 55.38	− 129.00 ± 44.74	111.00 − 318.00	38.00 − 188.00
Number of checks	4.71 ± 1.38	3.43 ± 2.23	− 1.29 ± 2.43	3.00 − 7.00	2.00 − 8.00
Degree of reduction	92.38 ± 2.29	94.45 ± 2.66	2.07 ± 3.19	89.59 − 94.86	89.98 − 97.93
Score	597.99 ± 20.70	900.76 ± 35.46	302.77 ± 43.66	562.74 − 627.36	846.13 − 955.55

The column *improvement* represents the difference between the results of both cases

**Table 2:** Statistical results regarding detailed components of the score

	Unit	Average ± SD		Range	
		Case 1	Case 2	Case 1	Case 2
Capitulum	Millimeters	0.62 ± 0.23	1.28 ± 0.83	0.25 − 0.91	0.47 − 2.75
Trochlea	Millimeters	1.92 ± 1.14	1.37 ± 0.81	0.62 − 4.06	0.58 − 2.90
Head	Millimeters	2.29 ± 2.22	0.87 ± 0.50	0.33 − 5.85	0.36 − 1.78
Lateral epicondyle	Millimeters	0.83 ± 0.52	1.63 ± 0.74	0.09 − 1.65	0.88 − 2.88
Medial epicondyle	Millimeters	0.93 ± 0.51	1.99 ± 0.30	0.41 − 1.83	1.65 − 2.44
Bicipital groove	Millimeters	2.74 ± 2.15	2.04 ± 2.02	0.96 − 5.92	0.66 − 6.58
Angle HSA	Degrees	1.27 ± 0.67	2.15 ± 0.66	0.63 − 2.41	1.41 − 3.32
Angle ECA	Degrees	1.32 ± 0.89	2.96 ± 0.58	0.25 − 3.02	2.50 − 4.03
Angle FEA	Degrees	2.26 ± 1.29	1.43 ± 1.10	0.63 − 3.96	0.51 − 3.07
Angle FEA-MED	Degrees	1.38 ± 1.09	1.37 ± 1.17	0.30 − 3.10	0.39 − 3.91
FE length	Millimeters	1.61 ± 1.50	0.40 ± 0.38	0.19 − 4.40	0.09 − 1.05
Humeral length	Millimeters	2.35 ± 2.08	0.83 ± 0.44	0.56 − 5.75	0.19 − 1.63
Distal Müller cube overlapping	Percentage	0.96 ± 0.02	0.96 ± 0.02	0.93 − 0.98	0.92 − 0.98
Proximal Müller cube overlapping	Percentage	0.92 ± 0.06	0.95 ± 0.03	0.83 − 0.97	0.89 − 0.98
Area of the rotation triangle	Millimeters^2^	0.01 ± 0.01	0.00 ± 0.00	0.00 − 0.03	0.00 − 0.01
ρ coefficient		0.92 ± 0.023	0.94 ± 0.027	0.90 − 0.95	0.90 − 0.98

Each row represents the difference of each metric between healthy and fractured side

After the experimental sessions were finished, all users rated the usability of the application with a 5, being this value *very easy* in a 1–5 Likert scale. The simulator was considered very intuitive, since its average score was a as 4.57 on the same scale. 70% of users have declared that they have prior experience using virtual reality in a medical context.

The use of an HTC Vive headset presents a comfortable choice for a high-quality virtual reality scenario, having a great immersion. It has adjustable straps, face cushion to reduce pressure and adaptive lenses. In addition, the HMD has enough space for users' glasses. As a result, it was considered comfortable (rated a 4.71 on average) and does not limit the movement of the individuals (a 4.57 out of 5). All users have rated the sharpness/fusion of the images as *correct* or *almost correct*. Finally, the isolation of the environment was qualified over a 4, *almost total immersion*, in all cases.

We have developed the simulator focusing on visual quality, since we tried to precisely replicate an operating room. Therefore, the users rated the realism of the room as 4.86 on average. Although a high-quality illumination, detailed meshes and post-processing corrections were employed, the performance was excellent (constantly surpassing 90 frames per second, the minimum recommended by the manufacturer). This is a crucial aspect to avoid sickness [46]. In fact, the fluidity of the movements averaged a 4.29 and no users have suffered dizziness during the test.

Once the training system has been satisfactorily evaluated, a future study can fulfill the requirements needed to include it in the intervention protocol of real surgeries. Therefore, it is expected to increase the feeling of security of the practitioners as well as to clarify their decision making, thus improving the marks of the interventions.

One of the main differences between the real and simulated environment is the absence of muscles and blood vessels in the virtual replica. However, since the goal is to train the sequence of steps to obtain a reduction that minimizes malrotation, having specific training will help better in that particular aspect. In further studies, specific exercises involving muscles and blood vessels can be incorporated depending on the suggestions of surgeons after including the training phase in the protocol.

## Conclusions

We have developed a simulator for training the surgical reduction of supracondylar humerus fractures. By emphasizing the movement of bone fragments, specialists acquire motor skills and knowledge to avoid the well-known malrotation of MIPO-treated fractures [9]. The implemented interaction paradigm represents a precise option to place the fragments in their proper anatomical position, as the user is allowed to grab each one with two hands. Furthermore, regarding the visual quality of the system, the designed scenario offers a realistic experience during the surgery, by employing high-quality illumination, post-processing effects and detailed medical assets.

An experimental session has been carried out to analyze the effectiveness of our proposal, producing promising results. We have observed a high learning rate when using the system. More concretely, the users have employed much less time to reduce the fracture in their second attempt. The individual indicators reveal that after training, the typical malrotation of MIPO is maintained below three degrees on average. Consequently, it is expected to minimize the risk of the patient during the intervention as well as to improve the accuracy of the reduction. In the future, we will extend this study, by monitoring the performance of the users in a real surgery and comparing it with the gathered results, in order to assess the actual improvement at the operating room.

Finally, our perspective is to enrich the system by including more steps of the intervention; cutting tissues, fixing the fracture with a plate, etc. Some constraints regarding vessels and other relevant tissue can be taken into account to restrict invalid movements. Moreover, the system can be adapted to other long bones which are likely to employ MIPO approaches.
